# The Silent Rejection: The Indirect Path From Partner Phubbing to Psychological Well‐Being Through Self‐Esteem and Romantic Loneliness

**DOI:** 10.1002/jcop.70146

**Published:** 2026-09-07

**Authors:** Muhammet Can Doğru, Tuğba Türk Kurtça

**Affiliations:** ^1^ Department of Psychological Counseling and Guidance Yıldız Technical University İstanbul Türkiye; ^2^ Department of Psychological Counseling and Guidance Trakya University Edirne Türkiye

**Keywords:** partner phubbing, psychological well‐being, romantic loneliness, self‐esteem, young adults

## Abstract

Romantic relationships play an important role in individuals' psychological well‐being. However, with the widespread use of digital technologies, these relationships may face new challenges. Partner phubbing refers to an individual's preoccupation with their phone during face‐to‐face interactions with a romantic partner and may weaken relational bonds. In this context, it is important to examine how this form of digital disengagement in romantic relationships is associated with individuals' psychological well‐being, as well as the mediating variables that may help explain this relationship. The study included 1627 heterosexual young adults (821 females, 806 males; age range = 18–35, *M* = 21.63) recruited through convenience sampling. The findings indicated that partner phubbing was associated with lower self‐esteem and higher levels of romantic loneliness. In the serial mediation model, self‐esteem and romantic loneliness fully mediated the relationship between partner phubbing and psychological well‐being. Identifying the mediating roles of self‐esteem and romantic loneliness may help clarify how partner phubbing is associated with individuals' internal evaluations and relationship experiences. These findings may contribute to the development of targeted interventions in clinical and counseling practice by illustrating how communication problems in romantic relationships affect psychological well‐being as part of broader social interaction patterns.

## 1 Introduction

Smartphones, as widely used personal communication tools, have contributed to a significant transformation in digital communication by facilitating interaction between individuals. These devices enable users to remain connected with their social environment at any time and from any location. However, recent studies suggest that this constant accessibility may also be associated with certain negative outcomes. In particular, intensive smartphone use has been linked to reduced quality of face‐to‐face social interactions as well as adverse psychological and physical outcomes (Garrido et al. 2021). Vanden Abeele et al. (2019), for example, examined face‐to‐face conversations among university students in dining areas and observed that more than 60% of conversations involved at least one instance of mobile phone use within a 10‐min interaction period. These findings highlight the pervasive presence of smartphones in everyday social interactions and their potential relevance for interpersonal communication dynamics. Phubbing refers to the use of a mobile phone during face‐to‐face communication while disengaging from the interpersonal interaction (Karadağ et al. 2015). When this behavior occurs within romantic relationships, it is referred to as partner phubbing (Roberts and David 2016).

Excessive smartphone use and partner phubbing within romantic contexts have been associated with a range of relational outcomes, including reduced communication quality, increased relational conflict, and lower relationship satisfaction (Krasnova et al. 2016). Empirical findings from cross‐sectional and experimental studies further suggest that partner phubbing is linked to feelings of exclusion, diminished self‐worth, and jealousy within romantic relationships (Chotpitayasunondh and Douglas 2018; McDaniel and Coyne 2016; Vanden Abeele et al. 2016). Longitudinal evidence further indicates associations between partner phubbing and lower relationship satisfaction through reduced intimacy within romantic relationships (Halpern and Katz 2017). These findings suggest that partner phubbing may be a relational behavior relevant to understanding variations in relationship functioning and relational well‐being in romantic contexts.

Within this framework, healthy romantic relationships are considered a central component of psychological well‐being, as higher relationship quality, partner support, and intimacy are associated with better psychological well‐being and lower clinical risk (Navaneetham and Kanth 2022; Komnik 2024). Examining partner phubbing in relation to psychological well‐being may contribute to a more integrated understanding of how digital relational behaviors are linked with both interpersonal functioning and well‐being outcomes.

Psychological well‐being is defined as an individual's capacity to cope with basic life tasks such as giving meaning to life, continuing personal development, and establishing satisfying relationships with others (Keyes et al. 2002). According to Ryff (1989) model, six dimensions are proposed: self‐acceptance, positive relations with others, autonomy, environmental mastery, purpose in life, and personal growth. Healthy romantic relationships represent a central component of the “positive relations with others” dimension, which reflects individuals' capacity to form close, trusting, and satisfying bonds (Yöyen et al. 2025). From this perspective, partner phubbing may be associated with disruptions in perceived relational support and commitment, which are directly relevant to psychological well‐being (Roberts and David 2016). Consistent with previous literature, phubbing behavior appears to be negatively associated with psychological well‐being (Al‐Saggaf 2023; Garrido et al. 2024; Zonash Mir 2020). However, the mechanisms underlying this association remain less clearly understood. Partner phubbing may be related to psychological well‐being both directly and indirectly through relational and individual factors. Self‐esteem and romantic loneliness are therefore proposed as potential mechanisms, which are further examined in the subsequent sections of this study.

### 1.1 Self‐Esteem and Romantic Loneliness as Mediators

Self‐esteem refers to an individual's evaluation of their own worth and reflects the extent to which they perceive themselves as valuable, capable, and acceptable (Baumeister et al. 2003; Wall 1986). Social experiences play an important role in the development of self‐esteem, as individuals' perceptions of acceptance, respect, and support from others contribute to how they evaluate themselves (Sevim and Artan 2021). Romantic relationships are particularly relevant in this regard because they represent a significant source of interpersonal validation and acceptance during young adulthood (Luciano and Orth 2017). In this regard, negative communication patterns within close relationships may be associated with lower self‐esteem. For example, individuals report feeling less prioritized and less valued when smartphone use interferes with time spent together with their romantic partner (Roberts and David 2016). Similarly, phubbing has been linked to threats to self‐esteem, belongingness, and existential meaning (Chotpitayasunondh and Douglas 2018).

Self‐esteem is also considered an important component of psychological functioning and psychological health (Crocker and Park 2004). Lower self‐esteem has been associated with a range of psychological difficulties (Weber et al. 2023), whereas higher self‐esteem is associated with healthier interpersonal relationships and greater confidence in oneself and others (Satir 1988). As such, self‐esteem may be viewed as both an indicator and a potential resource associated with psychological well‐being. Consistent with this perspective, previous studies have reported negative associations between phubbing and self‐esteem (Al‐Saggaf 2023; Bitar et al. 2021; Dai et al. 2024; Wang et al. 2021), whereas self‐esteem has been positively associated with psychological well‐being (Lee 2022; Poudel et al. 2020; Rehman et al. 2023).

Self‐esteem mediates the relationship between partner phubbing and psychological well‐being.

Weiss (1973) conceptualized loneliness in two broad categories: social and emotional loneliness. Emotional loneliness refers to the absence of sufficient emotional bonds with individuals with whom close relationships are expected, such as family members, romantic partners, or spouses (Weiss 1973). DiTommaso et al. (2004) further elaborated this concept and proposed that emotional loneliness consists of two subdimensions, namely romantic loneliness and family loneliness, making a significant contribution to the literature on emotional loneliness.

Romantic partners generally experience higher levels of emotional attachment and closeness when they are physically together (Parmaksız 2022). However, communication‐disrupting behaviors such as phubbing have been associated with reduced time spent together, lower perceived emotional support, and diminished feelings of closeness in romantic relationships (Krasnova et al. 2016). Consistently, previous studies have reported a positive association between phubbing and loneliness (Maftei and Măirean 2023; Taj et al. 2025; Zhan et al. 2022). In parallel, increased romantic loneliness has been associated with psychological difficulties such as depression, anxiety, and lower life satisfaction (Anyan et al. 2020), suggesting its relevance for psychological well‐being.

From the perspective of Ryff (1989) model, romantic loneliness may be particularly relevant to the “positive relations with others” dimension of psychological well‐being, which reflects individuals' capacity to form close, satisfying, and meaningful relationships. Difficulties in establishing emotional intimacy and maintaining relational satisfaction may be reflected in reduced perceived social connectedness (DiTommaso et al. 2004; Ryff 1989). Romantic loneliness may function as a relational experience associated with broader indicators of psychological adjustment. Consistent with this view, prior research has documented a negative association between loneliness and psychological well‐being (Camirand and Poulin 2022; Shpigelman and Vorobioff 2021; White et al. 2021).

Romantic loneliness mediates the relationship between partner phubbing and psychological well‐being.

### 1.2 Present Study

This study examines the association between partner phubbing and the psychological well‐being of young adults in romantic relationships, as well as the serial mediating roles of self‐esteem and romantic loneliness in this relationship. Partner phubbing, a modern communication problem, occurs when an individual prioritizes phone use over face‐to‐face interaction with their romantic partner, which may weaken relational bonds (Roberts and David 2016). Due to the multifaceted nature of psychological well‐being, it is important to understand how digital interactions relate to individual perceptions and relational experiences (Ryff 1989). The present research focuses on a theoretically informed process through which partner phubbing may be associated with self‐esteem, romantic loneliness, and psychological well‐being. At the conceptual level, partner phubbing is proposed to be related to individuals' self‐perceived value and, in turn, their perceptions of emotional connectedness within their romantic relationship.

The study is grounded in Vanden Abeele (2020) Attention–Arousal–Attribution Framework, which explains the social and psychological consequences of phubbing in everyday interpersonal interactions. Rather than directly testing this framework, the present study builds on and extends its sequential structure by applying it to individuals' experiences within romantic relationships. Specifically, the cognitive, emotional, and attributional processes proposed in the model are used to understand how individuals in romantic relationships may interpret and respond to perceived partner phubbing.

According to the framework, the process begins when an individual shifts attention from a social interaction to their partner's phone use. This attentional disruption is followed by negative emotional responses, including perceived social pain and feelings of ostracism (Vanden Abeele 2020). Rather than being a momentary interpersonal interruption, this experience may also activate fundamental psychological needs such as belonging, control, and self‐worth (Williams 2009). In the subsequent attributional stage, individuals engage in internal or external explanations to interpret this experience. Internal attributions involve self‐referential evaluations in which individuals interpret the cause of the experience in relation to their own relational value and self‐worth. Within this framework, self‐esteem reflects this immediate internal evaluative process, whereby individuals assess their perceived acceptance and relational value following exclusion (Leary and Baumeister 2000; Vanden Abeele 2020).

Importantly, this sequential ordering reflects the temporal structure of the Attention–Arousal–Attribution model, in which internal self‐evaluation processes precede broader relational appraisals. From this perspective, decreases in self‐esteem may represent the first cognitive outcome of perceived exclusion (Zwolinski 2012). Following this internal evaluation, individuals may then extend their interpretation from the self to the relationship context. In this stage, decreased self‐esteem may be associated with increased romantic loneliness, reflecting a perceived lack of emotional connection and intimacy with one's partner. This transition represents a shift from internal self‐evaluation processes toward relational appraisal processes (Vanden Abeele 2020). In other words, while self‐esteem reflects individuals' internal perception of relational worth, romantic loneliness reflects their subjective evaluation of emotional disconnection within the romantic relationship.

The adapted sequential structure suggests a theoretically informed pathway linking perceived partner phubbing with psychological well‐being. Within this pathway, partner phubbing is associated with self‐esteem, which in turn is associated with romantic loneliness and ultimately relates to psychological well‐being, including reduced life satisfaction and increased psychological distress (Vanden Abeele 2020).

Self‐esteem and romantic loneliness have a serial mediating role in the relationship between partner phubbing and psychological well‐being.

The present study contributes to both theoretical and applied discussions on digital interactions in romantic relationships. Beyond individual and dyadic processes, partner phubbing can also be situated within the wider social environments in which everyday digital interactions take place. From a community psychology perspective, attending to these environments may provide a more comprehensive understanding of how digital behaviors are associated with individual and relational well‐being. Previous research has documented associations between phubbing and self‐esteem (Dai et al. 2024), loneliness (Taj et al. 2025), and psychological well‐being (Garrido et al. 2024). However, much of this research has focused on phubbing as a general social behavior rather than on partner phubbing within romantic relationships. Moreover, although loneliness has been examined in relation to phubbing, limited attention has been given to romantic loneliness, which reflects the perceived absence of emotional closeness and intimacy in romantic relationships and may therefore be particularly relevant in the context of partner phubbing. By focusing on partner phubbing and examining the serial mediating roles of self‐esteem and romantic loneliness, the study extends the literature on digital relationship experiences and provides a relationship‐specific perspective on the associations between partner phubbing and psychological well‐being.

From a developmental perspective, the present study focuses on young adults, a developmental period characterized by identity exploration and increasing engagement in romantic relationships (Arnett 2000). According to Erikson's psychosocial development theory, this period corresponds to the “intimacy versus isolation” stage, in which the development of close and meaningful relationships is considered an important developmental task (Erikson 1968). Difficulties in establishing satisfying relational experiences during this period may be associated with lower self‐esteem and increased loneliness, which in turn are relevant to psychological well‐being (Camirand and Poulin 2022). Young adults also represent a particularly relevant group in this regard, as they are among the most intensive users of digital media (Pew Research P R Center 2024) and may therefore be more frequently exposed to relational dynamics involving digital communication, such as partner phubbing. The findings of the present study may inform the development of interventions targeting relationship functioning and digital media use. Such interventions could be incorporated into relationship counseling and couples therapy, while digital media awareness initiatives may provide an additional avenue for promoting healthier interaction patterns. Understanding the links between digital device use and relational experiences may also help practitioners recognize relational difficulties that could be addressed through preventive psychological counseling and psychoeducational interventions.

## 2 Method

### 2.1 Participants and Procedure

The study was conducted with 1627 heterosexual young adults, 821 (50.5%) females and 806 (49.5%) males. Participants were reached by the convenience sampling method. Specifically, participants were primarily recruited from a university context. Announcements containing the study link were disseminated via online platforms commonly used by university students (e.g., student networks and social media channels). Individuals who met the inclusion criteria and expressed interest voluntarily accessed the survey link and completed the online questionnaire. No course credit or financial compensation was provided for participation, and the researchers did not have any direct instructional or evaluative relationship with the participants. Inclusion criteria of the study: Being between the ages of 18–35, being in a romantic relationship and not being married. The mean age of the participants was 21.63 years (SD = 2.94). In terms of education level, 11 (0.7%) of the participants were basic education graduates, 215 (13.2%) were high school graduates, 1007 (61.9%) were university students, 366 (22.5%) were university graduates, and 28 (1.7%) were postgraduates. When daily social media usage was assessed, 17.8% (289) of participants reported using it for 0–2 h, 41.1% (669) for 2–4 h, 29.5% (481) for 4–6 h, 9.0% (146) for 6–8 h, and 2.6% (42) for 8 h or more. In addition, 1189 (73.1%) participants reported using their phones during meal times, 1572 (96.6%) participants reported using their phones during sleeping hours, and 620 (38.1%) participants reported checking their phones during face‐to‐face conversations with their partners.

The research data were collected online. After obtaining permission, the scales were transferred to Google Forms. In this process, the participants were informed using the informed consent form and the process was conducted with voluntary participants who agreed to participate. It took approximately 10 min to complete the form. No information was requested that would reveal the identity of the participants. To prevent data loss, it was mandatory to answer the questions on Google Forms. All procedures performed in this study were approved by the ethics committee of the relevant institutional review board and were conducted in accordance with the ethical standards of the Declaration of Helsinki.

### 2.2 Measures

#### 2.2.1 Partner Phubbing Scale

The scale aiming to measure participants' exposure to partner phubbing was developed by Roberts and David (2016). In the original scale, nine items are collected under a single factor. When Topal et al. (2021) adapted the scale into Turkish, three items were removed due to factor loadings and six items (e.g., “My partner glances at his/her cell phone while talking to me”) were grouped under a single factor in the adapted form. The scale is a 5‐point Likert scale ranging from 1 (never) to 5 (always). There are no reverse items. Rising scores indicate an increase in exposure to partner phubbing. In the adaptation study, Cronbach's alpha coefficient was 0.85 (Topal et al. 2021). It was also calculated as 0.84 in this study.

#### 2.2.2 Rosenberg Self‐Esteem Scale

The “self‐esteem” sub‐form of the Rosenberg Self‐Esteem Scale developed by Rosenberg (1965) was used. Turkish adaptation of the scale was made by Çuhadaroğlu (1986). The scale is a 4‐point Likert‐type scale ranging from 1 (very wrong) to 4 (very right). It consists of 10 items (e.g. “I think I am a valuable person”). Items 3, 5, 8, 9, and 10 are reverse scored. Increasing the scores obtained from the scale means increasing the level of self‐esteem. In the adaptation study, the validity coefficient was 0.71 and Cronbach's alpha coefficient was 0.75 (Çuhadaroğlu 1986). In this study, it was determined as 0.82.

#### 2.2.3 Social and Emotional Loneliness Scale (Selsa‐S)

The scale developed by DiTommaso et al. (2004) consists of 15 items. It includes three subscales: social loneliness subscale, emotional loneliness family subscale, and emotional loneliness romantic subscale (DiTommaso et al. 2004). The Turkish adaptation study was conducted by Akgül (2020). The scale preserved its structure in the original version. The scale items are 7‐point Likert type, ranging from 1 (strongly disagree) to 7 (strongly agree). In this study, the romantic loneliness subscale was used (e.g., “I have a lover/partner with whom I share my most intimate thoughts and feelings.”). The items were reverse‐coded where necessary, and higher scores indicate higher levels of romantic loneliness (Akgül 2020). The internal consistency coefficient of the scale for this study is 0.67.

#### 2.2.4 Psychological Well‐Being Scale

Diener et al. (2010) developed the scale to determine the psychological well‐being levels of individuals. Telef (2013) conducted the Turkish validity and reliability study of the scale. The scale consists of eight items (e.g., “I lead a purposeful and meaningful life”) and is a 7‐point Likert scale (Strongly disagree = 1, Strongly agree = 7). The scale items are grouped under a single factor. Increasing scores indicate that the level of psychological well‐being increases (Telef 2013). The internal reliability coefficient for this study was 0.80.

### 2.3 Data Analysis

During the study's analysis process, the descriptive statistics of the variables were first calculated, and then the correlations between them were examined. Next, two‐stage structural equation modeling (SEM) was applied. SEM is a method of analysis that allows one to test a theoretical model and evaluate causal relationships between variables (Kline 2015). First, the measurement model was tested, and then the structural model. The measurement model examined the relationships between latent and observed variables, while the structural model evaluated the causal paths between latent variables and the model's fit indices. The following goodness‐of‐fit indices and accepted thresholds were used to determine the model's validity: χ^2^/df ≤ 5, GFI, CFI, NFI, IFI, and TLI ≥ 0.90, and SRMR and RMSEA ≤ 0.08 (Hoyle and Panter 1995). When comparing mediation models, the AIC and ECVI values were considered, and models with lower values were deemed more appropriate (Akaike 1987). Since all study variables demonstrated unidimensional structures, item parceling was applied to reduce random measurement error and improve indicator reliability and distributional properties (Little et al. 2002). For each variable, two parcels were created by randomly distributing the items. Finally, the bootstrapping method was applied to test the direct and indirect effects between the variables.

## 3 Results

### 3.1 Preliminary Analysis

Descriptive statistics and correlation analysis findings of the variables are given in Table 1. It was observed that the skewness values of the variables ranged between −1.186 and 0.641, and the kurtosis values ranged between 0.047 and 2.965. According to Browne and Cudeck (1993), skewness values between −3 and +3 and kurtosis values between −10 and +10 are sufficient to ensure normality. In this context, the results obtained are within these acceptable ranges, indicating that all variables have normal distribution characteristics.

**Table 1:** Descriptive statistics and correlations.

	Descriptive statistics				Correlations		
Variables	*M*	SD	Skewness	Kurtosis	1	2	3
1. Partner phubbing	13.14	4.15	0.641	0.110	—		
2. Self‐esteem	32.97	5.67	−0.830	0.047	−0.083**	—	
3. Romantic loneliness	10.35	5.23	0.867	0.273	0.261**	−0.255**	—
4. Psychological well‐being	44.55	6.46	−1.186	2.965	−0.130**	0.511**	−0.231**

** *p* < 0.001.

### 3.2 Measurement Model

After testing the measurement model, which consisted of four latent constructs (partner phubbing, self‐esteem, romantic loneliness, and psychological well‐being) and eight observed variables, it was determined that the model fit the data well [*χ* ^*2*^_(14, 1627)_ = 23.90; *χ* ^*2*^/df = 1.71, *p* < 0.05; GFI = 0.99; CFI = 0.99; NFI = 0.99; IFI = 0.99; TLI = 0.99; SRMR = 0.01; RMSEA = 0.02]. All factor loadings were found to be between 0.70 and 0.92 at a significant level (*p* < 0.001). These findings suggest that the observed variables accurately reflect their respective latent constructs.

### 3.3 Structural Model

**Figure 1:**
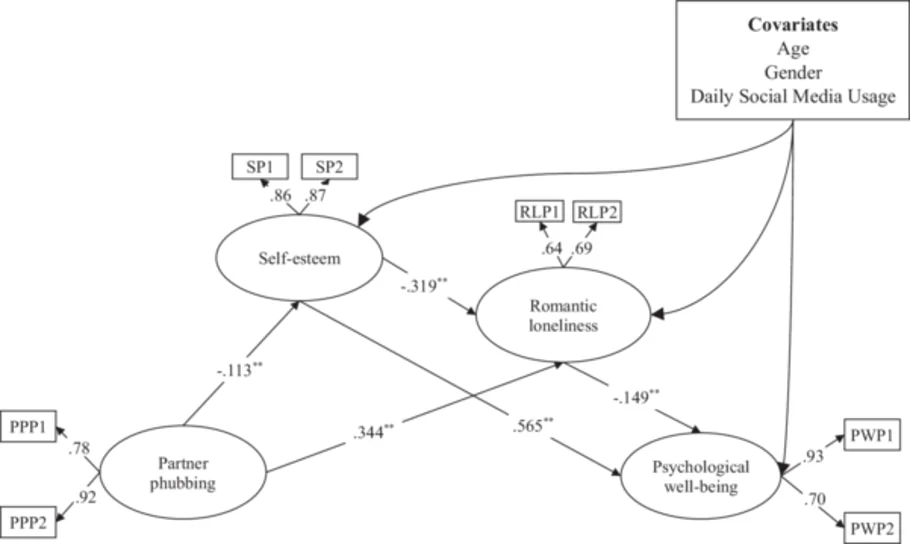
Structural equation modeling for the serial mediation model. **p < 0.001. PPP, parcel of partner phubbing; PWP, parcel of psychological well‐being; RLP, parcel of romantic loneliness; SP, parcel of self‐esteem.

First, a fully mediated model was tested in which the relationship between partner phubbing and psychological well‐being was represented only through indirect pathways. The fit indices of this model show that the model fits the data well: [*χ* ^*2*^_(33, 1627)_ = 118.64; *χ* ^*2*^/df = 3.59, *p* < 0.001; GFI = 0.99; CFI = 0.98; NFI = 0.98; IFI = 0.98; TLI = 0.97; SRMR = 0.03; RMSEA = 0.04]. After this stage, the partially mediated model including the direct relationship between partner phubbing and psychological well‐being was analyzed. However, the direct path was not significant (*β* = −0.039, *p* > 0.05). Given the lower AIC and ECVI values, acceptable fit indices, and the non‐significant direct path, the fully mediated model was considered the better‐fitting model. The findings suggest that the relationship between partner phubbing and psychological well‐being may be indirectly associated with self‐esteem and romantic loneliness. The findings related to this model are visualized in Figure 1.

### 3.4 Bootstrapping

Bias‐corrected bootstrapping with 5000 resamples was used to examine the indirect associations in the proposed model. The standardized indirect coefficients and their 95% confidence intervals are presented in Table 2. The results indicated a significant indirect association between partner phubbing and psychological well‐being through self‐esteem (bootstrap coefficient = −0.137, 95% CI = −0.215, −0.062). In addition, romantic loneliness was significantly associated with the relationship between partner phubbing and psychological well‐being through an indirect pathway (bootstrap coefficient = −0.109, 95% CI = −0.173, −0.055). Furthermore, the indirect association between partner phubbing and psychological well‐being through the sequential pathway of self‐esteem and romantic loneliness was also statistically significant (bootstrap coefficient = −0.011, 95% CI = −0.022, −0.005).

**Table 2:** Indirect effects for the mediational model.

		95% CI	
Model pathways	Estimated	Lower	Upper
Partner phubbing→Self‐esteem→Psychological well‐being	−0.137**	−0.215	−0.062
Partner phubbing→Romantic loneliness→Psychological well‐being	−0.109**	−0.173	−0.055
Partner phubbing→Self‐esteem→Romantic loneliness →Psychological well‐being	−0.011**	−0.022	−0.005

** *p* < 0.001.

## 4 Discussion

This study aimed to provide a holistic model by examining the association between perceived partner phubbing and psychological well‐being through self‐esteem and romantic loneliness. As technological devices have become increasingly integrated into everyday life, research has suggested that behaviors such as partners frequently turning their attention to their phones during interactions are associated with feelings of worthlessness, exclusion, and loneliness within romantic relationships (Roberts and David 2016). Romantic relationships constitute an important context in which individuals experience belonging, closeness, and support, and are therefore closely linked to psychological well‐being (Gómez‐López et al. 2019). Furthermore, focusing on young adults is particularly relevant because this developmental period is characterized by ongoing identity formation and the establishment of close interpersonal relationships (Arnett 2000). The present study contributes to the literature by examining the associations among perceived partner phubbing, self‐esteem, romantic loneliness, and psychological well‐being within the context of romantic relationships in the digital age.

First, self‐esteem was found to statistically mediate the association between partner phubbing and psychological well‐being, supporting H_1_, albeit based on a relatively modest association between partner phubbing and self‐esteem. To our knowledge, previous studies have not examined the mediating role of self‐esteem in the relationship between partner phubbing and psychological well‐being. However, previous research has consistently reported negative associations between phubbing and self‐esteem (Al‐Saggaf 2023; Barbed‐Castrejón et al. 2024; Xie et al. 2020). Furthermore, in one of the few studies examining this relationship within romantic relationships using a dyadic approach, partner phubbing was negatively associated with self‐esteem (Chmielik and Błachnio 2021). Prior research has also linked self‐esteem with higher levels of psychological well‐being (Rehman et al. 2023; Rippon et al. 2024). The present findings are consistent with the view that partner phubbing may be associated with lower self‐esteem, which may in turn be related to lower psychological well‐being. Within romantic relationships, a partner's frequent attention to their phone during face‐to‐face interactions may be interpreted as a signal of reduced relational value or acceptance. Such perceptions may shape individuals' evaluations of their social and relational worth, potentially contributing to lower self‐esteem. In sum, these findings highlight the relational nature of self‐esteem and suggest that it may represent a relatively weak but theoretically meaningful pathway linking perceived partner phubbing with psychological well‐being.

Second, romantic loneliness was found to mediate the association between partner phubbing and psychological well‐being, supporting H_2_. Although previous studies have not directly examined romantic loneliness as a mediator in this context, existing evidence indicates that phubbing in romantic relationships is positively associated with loneliness (Frackowiak et al. 2024; Zhan et al. 2022). Individuals exposed to higher levels of partner phubbing have been reported to experience greater feelings of exclusion and insignificance (Ligon‐Tucker 2023). Previous research has also consistently shown that loneliness is associated with lower levels of psychological well‐being (Dalal and Singh 2022; Xu 2024). Altogether, these findings are in line with an interpretation in which higher perceived partner phubbing is associated with greater romantic loneliness, which in turn is associated with lower psychological well‐being. However, these interpretations should be considered in light of the relatively modest internal consistency of the romantic loneliness subscale. From a theoretical perspective, the mediating role of romantic loneliness reflects the relevance of the need to belong, which posits that humans are motivated to form and maintain meaningful interpersonal bonds (Baumeister and Leary 1995). When this need is not sufficiently fulfilled, individuals may experience heightened loneliness and reduced psychological well‐being. Within romantic relationships, frequent attention to a partner's phone during face‐to‐face interactions may be associated with reduced perceived emotional closeness and a sense of relational distance. In this study, loneliness was conceptualized specifically as romantic loneliness rather than general loneliness, capturing relationship‐specific experiences of emotional disconnection. The present findings therefore contribute to the literature by highlighting how perceived partner phubbing may be linked to romantic loneliness and its potential implications for psychological well‐being.

Finally, self‐esteem and romantic loneliness were found to sequentially mediate the association between partner phubbing and psychological well‐being, supporting H_3_. The proposed serial mediation model is intended as a theoretically grounded, process‐oriented explanation rather than a causal pathway, in line with the cross‐sectional design of the study. Although the literature has not yet offered an integrated model that simultaneously considers these variables, prior research links phubbing with lower self‐esteem (Błachnio and Przepiorka 2018), self‐esteem with loneliness (Saricam et al. 2012), and loneliness with poorer psychological well‐being in romantic contexts (Özdoğan 2021). Taken together, these findings support the proposed sequential mediation model. Specifically, higher perceived partner phubbing is associated with lower self‐esteem, which is in turn associated with greater romantic loneliness and, subsequently, lower psychological well‐being. Vanden Abeele (2020) Attention–Arousal–Attribution framework provides a useful lens for understanding this ordering by conceptualizing phubbing as a social interruption that unfolds through attentional disruption, emotional arousal, and subsequent evaluative processes. From this perspective, the consequences of disrupted interpersonal attention may extend beyond immediate evaluations of the interaction to broader experiences of relational disconnection, which may be reflected in greater romantic loneliness. Thus, the sequential association between self‐esteem and romantic loneliness may provide a theoretically coherent account of how partner phubbing is indirectly related to psychological well‐being across interconnected relational and psychological processes.

Within this framework, partner phubbing can be understood as being linked to self‐referential evaluations of relational worth, reflected in self‐esteem. Lower self‐esteem may then be accompanied by heightened perceptions of emotional distance in the relationship, reflected in romantic loneliness. Romantic loneliness, in turn, relates to lower psychological well‐being through reduced perceived relational connectedness. The pattern observed in the present study is broadly consistent with a sequential reading of the Attention–Arousal–Attribution framework, although it should be interpreted within the limits of a cross‐sectional design. This interpretation also extends beyond the immediate romantic dyad. Partner phubbing may be understood not only as an interpersonal interaction pattern but also as a digital behavior shaped by the social environments in which individuals communicate and maintain relationships. This broader view draws attention to the ways in which everyday digital practices may intersect with relational experiences and individual psychological well‐being.

The findings of this study should be interpreted in light of its cross‐sectional design, which does not allow for the examination of causal relationships. While the mediation results are informative about potential associations among the variables, causal interpretations should therefore be avoided. Future longitudinal or experimental research would be useful to better capture how these variables may be related across time. The use of item parceling may also mask item‐level variability and influence parameter estimates; therefore, the results should be interpreted within this methodological context. The association between perceived partner phubbing and self‐esteem was relatively weak in the present study. A recent meta‐analysis suggests that partner phubbing tends to show stronger and more consistent associations with relational outcomes, such as relationship satisfaction, relationship quality, and emotional closeness, whereas its associations with individual‐level psychological variables, including self‐esteem, appear to be smaller and more variable across studies (Ni et al. 2025). These findings suggest that the associations between perceived partner phubbing and individual psychological processes may be more indirect and context‐dependent than direct in nature. The relatively small effect size should be considered when interpreting the mediating role of self‐esteem.

### 4.1 Limitations and Implications

This study has several limitations. First, because data were collected via self‐report measures, the possibility of response bias related to social desirability should be considered. This may be particularly relevant for variables involving personal and relational evaluations, such as self‐esteem and romantic loneliness. Future studies may benefit from incorporating alternative data sources such as partner reports, observational methods, or ecological momentary assessment (EMA), which can capture experiences in real time and reduce reliance on retrospective self‐reports.

Second, the cross‐sectional design of the study limits the extent to which directional or causal interpretations can be made regarding the associations among the variables. Future longitudinal or experimental research would be valuable for examining how these associations may unfold over time.

Third, the present study relied on individual‐level data. Given that romantic relationships are inherently dyadic, future research would benefit from adopting dyadic designs to better capture reciprocal processes between partners. In particular, examining partner phubbing from both individuals' perspectives may provide a more comprehensive understanding of attachment processes, loneliness, and psychological well‐being.

Fourth, the study sample was drawn from a convenience sampling approach and consisted of young adults who were largely university students, heterosexual, and unmarried. This limits the generalizability of the findings to broader populations, such as older adults, married individuals, or individuals with more diverse sexual orientations and relationship statuses. Therefore, future research should aim to replicate the present findings in more diverse and representative samples to enhance external validity.

Finally, the internal consistency of the romantic loneliness subscale was slightly below the conventional threshold (*α* = 0.67). This suggests that some degree of measurement error may be present, which could have attenuated the observed associations involving romantic loneliness. The mediation pathway involving romantic loneliness should therefore be interpreted with appropriate caution. However, given the theoretical importance of romantic loneliness in close relationships, the construct was retained and interpreted within a broader relational framework.

The findings suggest that partner phubbing is associated with individual technology use patterns as well as broader social psychological processes. These processes may involve how individuals perceive their social inclusion, evaluate themselves within relationships, and maintain their commitment to those relationships. This perspective provides a theoretically informed account of how digital relational behaviors may be linked to psychological well‐being processes in romantic relationships.

The study was conducted with young adults aged 18–35, a developmental period characterized by intensive engagement with mobile technologies and the intimacy versus isolation stage described in Erikson (1968) psychosocial development theory. During this developmental period, romantic relationships may be particularly sensitive to digital interaction patterns. The results may help clarify how digital behaviors are linked to self‐evaluative and relational processes during developmentally important stages. From an applied perspective, the findings may inform counselors, therapists, and clinicians working with romantic relationships. The findings may suggest that interventions could benefit from addressing not only communication patterns, but also areas such as digital attention management, relational awareness, self‐esteem enhancement, and emotional expectation management within romantic relationships.

From a community psychology perspective, partner phubbing can be understood within broader digital interaction contexts that shape everyday relational experiences. Repeated experiences of divided attention in romantic interactions may be associated with changes in individuals' relational perceptions and self‐evaluations, which are relevant to broader psychosocial adjustment processes. Understanding how digital behaviors are interpreted within close relationships may inform community‐based interventions. Psychoeducational programs could help individuals recognize and address the relational consequences of problematic digital behaviors. Digital well‐being initiatives could further promote healthier interaction patterns by increasing awareness of how everyday technology use may shape close relationships. Such approaches may support relationship quality, emotional connectedness, and perceived social inclusion in increasingly digitalized social environments.

## 5 Conclusion

This study examined the indirect associations between partner phubbing and individual psychological well‐being in romantic relationships and proposed a model including the sequential mediating roles of self‐esteem and romantic loneliness. Although the study has some limitations, the findings are valuable in terms of illustrating how relationship dynamics may be intertwined with individual psychological processes. The findings suggest that not only individuals' momentary experiences within romantic relationships, but also the interplay between these experiences, self‐perceptions, and emotional needs may be relevant for psychological well‐being. In particular, highlighting the role of romantic loneliness in the context of relational processes offers insight into how perceived relational disconnection may be linked to well‐being outcomes. In this regard, the proposed model may provide a useful framework for understanding how neglected behaviors in romantic relationships are associated with individuals' psychological health. Overall, the study offers a basis for further research on how digital forms of interaction may be linked to couple dynamics and individual well‐being processes.

## Author Contributions

Conceptualization, data curation, formal analysis, investigation, methodology, validation, visualization, writing – original draft, writing – review and editing: Muhammet Can Doğru and Tuğba Türk Kurtça. Supervision: Tuğba Türk Kurtça.

## Funding

The authors have nothing to report.

## Ethics Statement

Our research was conducted in accordance with the ethical standards. We obtained all necessary approvals and permissions to conduct the study, ensuring the privacy, confidentiality, and rights of the participants involved. Informed consent was obtained from all subjects involved in the study. The necessary permission for this study was obtained from Trakya University Social and Human Sciences Ethics Committee (Verification Code: BSUCMC4M6Z).

## Conflicts of Interest

The authors declare no conflicts of interest.

## Data Availability

The data from this study are available from the corresponding author upon request.
